# Analysis of qPCR reference gene stability determination methods and a practical approach for efficiency calculation on a turbot (*Scophthalmus maximus*) gonad dataset

**DOI:** 10.1186/1471-2164-15-648

**Published:** 2014-08-04

**Authors:** Diego Robledo, Jorge Hernández-Urcera, Rosa M Cal, Belén G Pardo, Laura Sánchez, Paulino Martínez, Ana Viñas

**Affiliations:** Departamento de Genética, Facultad de Biología (CIBUS), Universidad de Santiago de Compostela, 15782 Santiago de Compostela, Spain; Instituto Español de Oceanografía, Centro Oceanográfico de Vigo, 36390 Vigo, Spain; Departamento de Genética, Facultad de Veterinaria, Universidad de Santiago de Compostela, 27002 Lugo, Spain

**Keywords:** qPCR, Reference genes, Amplification efficiency, Turbot, *Scophthalmus maximus*, Gonad

## Abstract

**Background:**

Gene expression analysis by reverse transcription quantitative PCR (qPCR) is the most widely used method for analyzing the expression of a moderate number of genes and also for the validation of microarray results. Several issues are crucial for a successful qPCR study, particularly the selection of internal reference genes for normalization and efficiency determination. There is no agreement on which method is the best to detect the most stable genes neither on how to perform efficiency determination. In this study we offer a comprehensive evaluation of the characteristics of reference gene selection methods and how to decide which one is more reliable when they show discordant outcomes. Also, we analyze the current efficiency calculation controversy. Our dataset is composed by gonad samples of turbot at different development times reared at different temperatures. Turbot (*Scophthalmus maximus*) is a relevant marine aquaculture European species with increasing production in the incoming years. Since females largely outgrow males, identification of genes related to sex determination, gonad development and reproductive behavior, and analysis of their expression profiles are of primary importance for turbot industry.

**Results:**

We analyzed gene stability of six reference genes: *RPS4*, *RPL17*, *GAPDH*, *ACTB*, *UBQ* and *B2M* using the comparative delta-CT method, Bestkeeper, NormFinder and GeNorm approaches in gonad samples of turbot. Supported by descriptive statistics, we found NormFinder to be the best method, while on the other side, GeNorm results proved to be unreliable. According to our analysis, *UBQ* and *RPS4* were the most stable genes, while *B2M* was the least stable gene. We also analyzed the efficiency calculation softwares LinRegPCR, LREanalyzer, DART and PCR-Miner and we recommend LinRegPCR for research purposes since it does not systematically overestimate efficiency.

**Conclusion:**

Our results indicate that NormFinder and LinRegPCR are the best approaches for reference gene selection and efficiency determination, respectively. We also recommend the use of *UBQ* and *RPS4* for normalization of gonad development samples in turbot.

**Electronic supplementary material:**

The online version of this article (doi:10.1186/1471-2164-15-648) contains supplementary material, which is available to authorized users.

## Background

The main quantitative method for the study of gene expression is reverse transcription real-time quantitative PCR (qPCR), which is considered a highly sensitive technique. In qPCR, the amount of amplified product is monitored during the course of the reaction by measuring the fluorescence during the annealing phase of each amplification cycle. Fluorescence is produced by dyes or probes which bind to DNA, and so it is proportional to the amount of synthesized product. The DNA intercalating dye SYBR green I is one of the most widely applied systems, since the fluorescence readings can be obtained from any PCR amplicon, irrespective of its sequence [1]. Two types of qPCR can be performed: the expression levels of the genes can represent either an absolute quantification that relates the PCR signal to the initial copy number using a calibration curve or, as in our work, a relative quantification which measures the relative change in RNA expression level. A number of technical parameters such as RNA and cDNA quality, primer specificity, PCR efficiency and the genes used for normalization heavily condition the quality of qPCR results. Despite the widespread popularity of qPCR, there is a worrying lack of consensus on how it should be performed and how its results should be analyzed. The publication of the MIQE guidelines [2] represented a landmark towards qPCR standardization, but not only are there many publications which still ignore the MIQE guidelines, but also new controversies have arisen which require further discussion.

Due to the quantitative nature of qPCR, an appropriate normalization method is critical to achieve reliable results. The purpose of normalization is to remove sampling noise (such as RNA differences in concentration and its quality) in order to estimate gene expression accurately [3]. Ideally, reference genes used for this purpose should show the same level of expression in all cells and tissues, and remain stable under different experimental conditions. As pointed out in several publications, there is no universal reference gene, and housekeeping gene expression can vary considerably [4], the best reference gene probably varying in the same species according to the tissue and the experimental conditions [5]. So, as mentioned in the MIQE guidelines, normalization against a single reference gene is not recommended unless a clear evidence of its invariant expression is described for the specific experimental conditions of the study. The optimal number and choice of reference genes should be experimentally determined [2], yet many publications employ a single normalization gene without appropriate validation. Several methods and software have been described to determine the optimum reference genes, however which method is the most suitable has still not been addressed.

Four reference gene determination methods are commonly used in qPCR studies: the comparative delta-Ct method [6], BestKeeper [7], Genorm [3] and NormFinder [8]. Gene expression stability is evaluated differently in each of the four methods. Briefly, the comparative delta-Ct method calculates the stability of each gene by obtaining the standard deviation of Cq differences (Cq or quantification cycle is the number of amplification cycles required to reach a selected fluorescence threshold) within each sample for each pairwise comparison with the other genes and averaging them. NormFinder takes into account both intra-group and inter-group gene variation to evaluate its stability. BestKeeper ranks the genes according to the standard deviation (SD) of their Cqs, but the output includes more information, for example the coefficient of variation (CV), which was proposed as a validation method for the results offered by NormFinder and GeNorm [9]. GeNorm determines the pairwise standard deviation of Cq values of all genes, and then excludes the one with the lowest stability, repeating the process until only two genes remain, which are then considered the most stable ones.

Another topic, which has recently focused the attention of specialist on this ground, is the kinetics of qPCR and the efficiency determination associated to it. Traditionally, standard curves have been the gold standard to calculate qPCR efficiency. However, pipetting errors or poorly calibrated pipettes can greatly affect the accuracy of the standard curves due to the cumulative nature of error [10, 11]. Also, cDNA may include PCR inhibitors which diminish the efficiency of the qPCR reaction. These inhibitors often remain in the samples from steps prior to qPCR amplification. The dilution steps involved in standard curve construction, which also dilute inhibitors, might lead to efficiency overestimation [10]. This can be easily confirmed by the existence of efficiencies above 100% and the usual practice of accepting a pair of primers as valid if its efficiency is between 90-110%. Theoretically, it is impossible to obtain qPCR efficiencies above 100%. More recently, several mathematical models have been published describing the kinetics of the qPCR reaction and trying to estimate qPCR efficiency from a single reaction. Many different models have been proposed, ranging from exponential [10, 12] to logistic ones employing up to five parameters [1]; even more complex models, which take into account the efficiency of each of the steps of the qPCR reaction, have been tackled [13]. Here we analyzed four methods which allow an easy determination of efficiency for each reaction and amplicon: i) LinRegPCR [14], ii) LREanalyzer [15], iii) DART [10] and iv) PCR-Miner [16], all publicly available and implemented in user-friendly software or online applications.

Marine flatfish represent a valuable group of teleosts because of their highly appreciated white flesh [17]. Turbot is a marine flatfish species with a notable aquaculture projection in Europe. It is predicted that by 2014 its production will duplicate that of 2009 (9142 t) (FEAP). Also, since turbot was introduced in China in 1992, the farming industry of this species has developed into one of the main mariculture industries with a production of 50000 tons per year [18]. The main trait targets for genetic breeding programs in this species are growth rate, sex ratio and disease resistance [19]. Turbot shows one of the largest sex-dependant size dimorphism in marine aquaculture [20]: females outgrow males by 50% when they reach commercial size. Some studies have demonstrated a ZZ/ZW system in turbot [21, 22] and identified the main sex determining region in linkage group (LG) 5 [22], but these authors also suggest the existence of other minor genetic and environmental factors, for example temperature, which might affect sex determination. However, expression analyses have only been carried out in immune tissues so far [23–25]. Reference genes for qPCR have been characterized in different tissues of turbot [5] and in other flatfish [26, 27], but gonads have not been included in these studies.

In this study, we evaluated the main factors which might compromise qPCR results, reference gene choice and qPCR efficiency determination, using gonads of turbot reared at different temperatures and along the development process. Our results suggest that for research purposes, NormFinder and LinRegPCR implement the best approaches for reference gene selection and efficiency determination, respectively, ant that *UBQ* and *RPS4* would be the best reference genes for the normalization of gonad development in turbot from 30 up to 135 days post fertilization. To our knowledge, this is the first qPCR evaluation in turbot gonads and no similar studies have been carried out in fish to date. Our approach, although applied in a particular tissue in turbot could be used as a guideline for qPCR development in other tissues or species.

## Results

### Amplification

Amplification of each reference gene in 240 samples (two replicates per sample) produced a 480 Cq values dataset. Samples with missing Cq values or inconsistencies between replicates (Cq differences >1 cycle) in any of the reference genes were removed from the analysis. After averaging duplicates a total of 212 samples were kept (28 samples were removed) and we obtained descriptive statistics and Kolmogorov-Smirnov tests to check for normality for each of the assayed genes (Table 1). A single amplification product for each primer pair was confirmed by a single peak in the melting curve analysis and also by PCR product sequencing.

**Table 1:** **Descriptive statistics of the reference genes Cq values**

Gene	N	Mean	SD	Min Cq	Max Cq	KS-test p
***ACTB***	212	15.87	1.21	13.52	19.02	0.197
***B2M***	212	19.50	1.70	16.51	24.44	0.009
***GAPDH***	212	20.18	1.65	16.81	24.72	0.477
***RPL17***	212	18.44	1.46	15.67	22.70	0.130
***RPS4***	212	21.21	1.65	17.82	25.43	0.739
***UBQ***	212	18.34	1.12	15.68	21.09	0.108

Number of samples (*N*), mean, Standard deviation (*SD*), Minimum Cq value (*Min Cq*), Maximum Cq value (*Max Cq*) and p value of the Kolmogorov-Smirnov test (*KS-test p*) are shown for each candidate reference gene.

*ACTB* showed the highest expression (Cq mean = 15.87), amplification being more than two cycles earlier than any other gene. On the other side, *RPS4* showed the lowest expression (Cq mean = 21.21). *UBQ* standard deviation (SD) was the lowest (1.12) while *B2M* presented the largest variation between Cq values (SD = 1.70). Also, reference gene Cq distributions were normal in every case but that of *B2M* (Kolmogov-Smirnov test p = 0.009).

**Figure 1:**
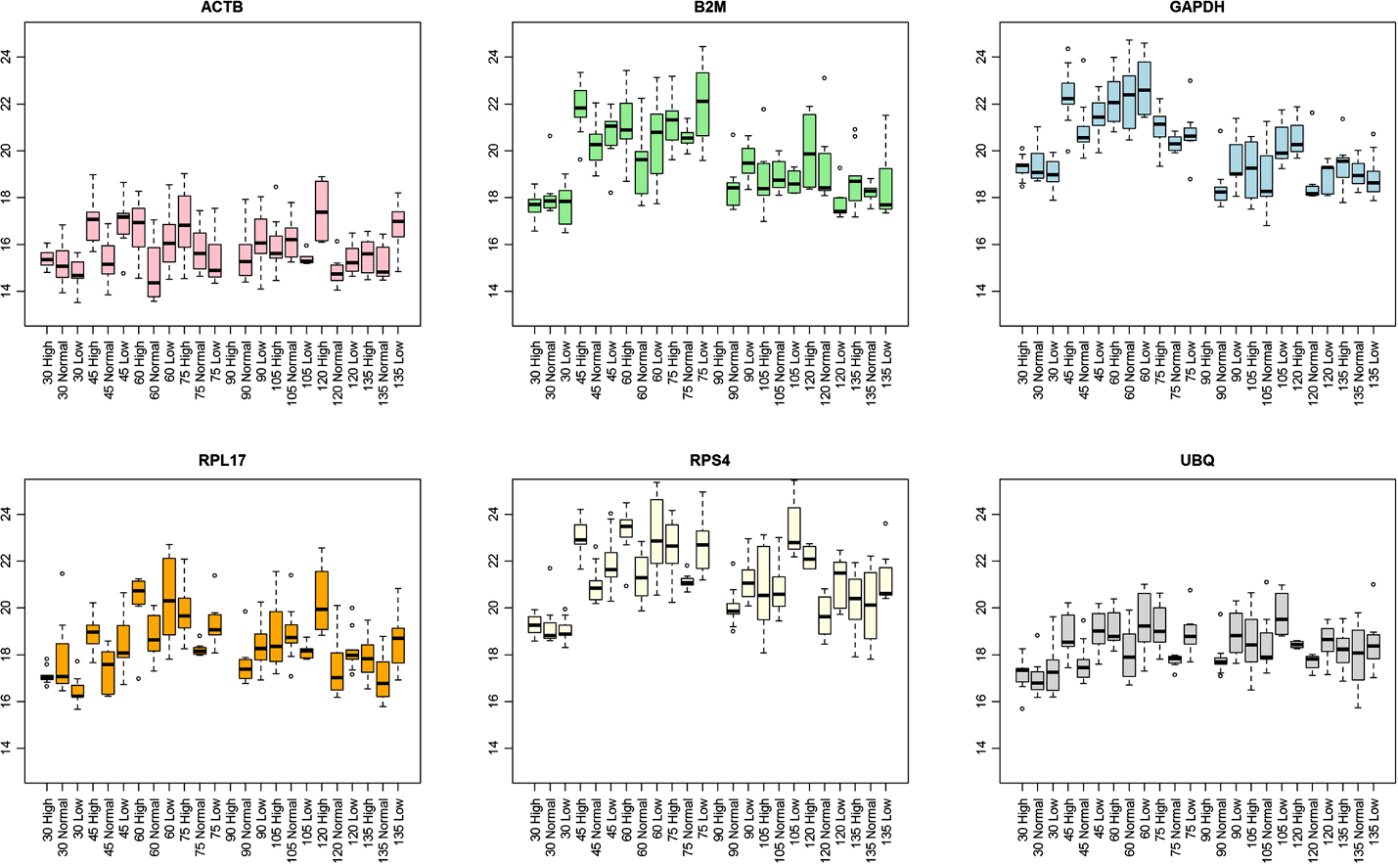
**Reference gene Cq value distributions.** Boxplots of the Cq values in each experimental group (fish age /temperature) for each of the six reference genes. Each group is named with a number, which indicates age in days post fertilization, and either “High”, “Normal” or “Low” which indicates rearing temperature.

According to the experimental design, samples were divided in groups according to fish age in days post fertilization (dpf) and rearing temperature. This produced a total of 24 groups (8 age groups × 3 temperatures), with a minimum of six samples per group and a maximum of ten. A boxplot of all the groups for the six reference genes can be observed in Figure 1. We also considered grouping our samples by degree-days, however, since groups remained basically the same (only two age-temperature groups would merge, so the number of groups would change from 24 to 23), which did not alter neither the results nor the discussion, we decided to name the groups by their age and rearing temperature since we considered it clearer (see Additional file 1: Tables S1 and S2).

A similar dataset of Cq values for six sex differentiation related genes was obtained and their descriptive statistics are presented in Table 2. These genes are involved in gonad differentiation and were used to check normalization and efficiency correction effects.

**Table 2:** **Descriptive statistics of the genes involved in gonad differentiation Cq values**

Gene	N	Mean	SD	Min Cq	Max Cq	KS-test p
***CYP19a***	224	31.87	5.35	20.44	40	0.003
***AMH***	224	26.34	2.84	19.75	40	0.000
***SOX19***	224	26.58	3.58	16.75	38.73	0.000
***SOX9***	224	24.77	1.98	21.24	30.89	0.000
***VASA***	224	26.17	4.25	16.78	35.90	0.000
***SOX17***	224	29.39	2.78	20.34	36.24	0.001

Number of samples (*N*), mean, Standard deviation (*SD*), Minimum Cq value (*Min Cq*), Maximum Cq value (*Max Cq*) and p value of the Kolmogorov-Smirnov test (*KS-test p*) are shown for each target gene.

### Analysis of the reference genes

**Figure 2:**
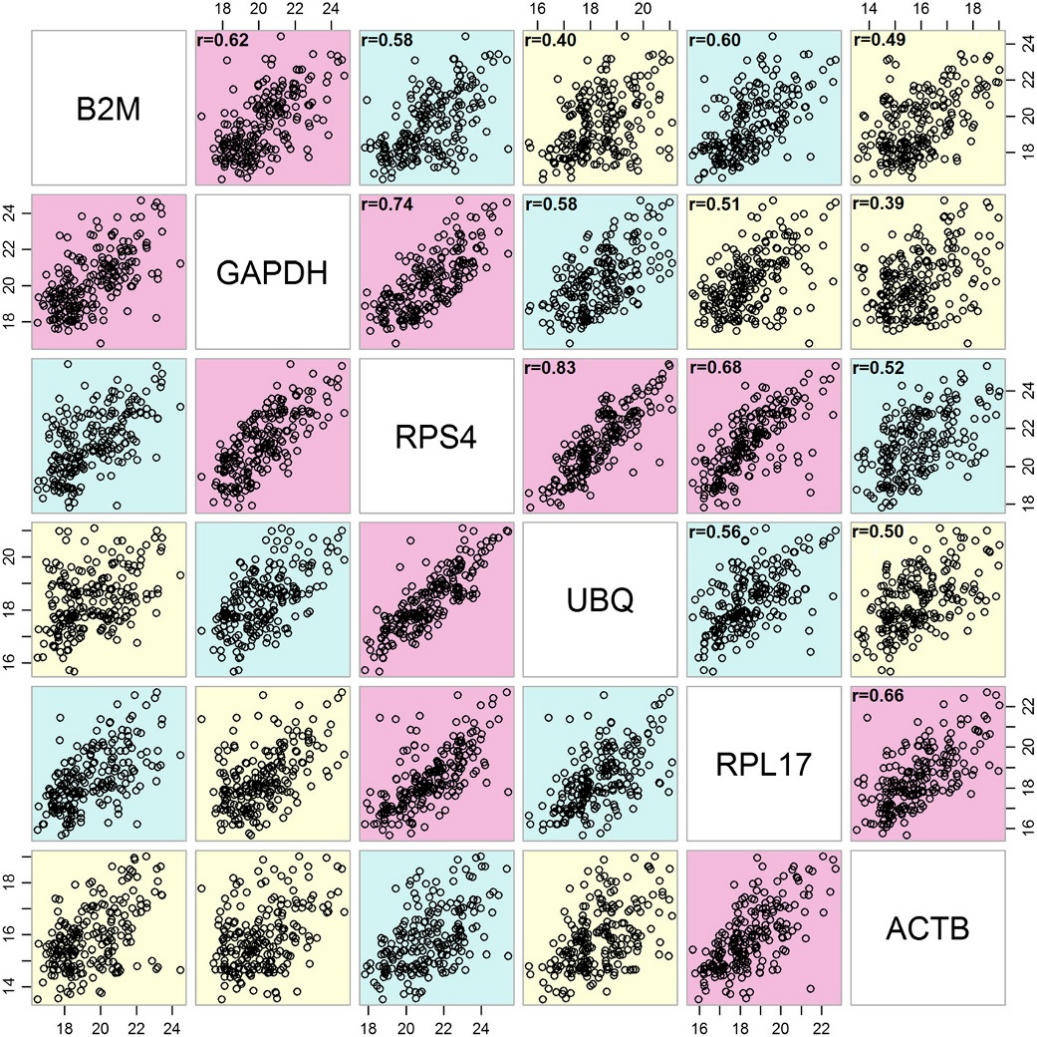
**Correlation between reference genes.** Legend: Correlation between reference genes Cq values. The highest correlations are colored in red, medium correlations in green and the lowest in yellow. Correlation coefficient (r) values are shown, p value < 0.001.

We analyzed the 212 Cq values obtained for each of the reference genes with comparative delta Ct method, Bestkeeper, NormFinder and GeNorm. For each method and gene a ranking of stability values is shown with the most stable gene at the top and the least stable at the bottom (Table 3). Due to the importance of gene-to-gene correlations for comparative delta-Ct method and GeNorm, correlations were graphically represented (Figure 2). Finally, the average intergroup and intragroup variation for each gene is shown in Table 4 as reported by NormFinder. Groups were formed as specified above according to fish age and rearing temperature.

**Table 3:** **Stability rankings obtained with the different reference gene determination methods**

Rank	Comparative Delta-Ct	BestKeeper (SD)	BestKeeper (CV%)	NormFinder	GeNorm
**1**	UBQ (1.267)	UBQ (1.12)	UBQ (4.96)	RPS4 (0.613)	UBQ/RPS4 (0.952)
**2**	RPS4 (1.278)	ACTB (1.21)	ACTB (6.16)	UBQ (0.713)	
**3**	RPL17 (1.323)	RPL17 (1.46)	RPL17 (6.34)	RPL17 (0.721)	RPL17 (1.154)
**4**	ACTB (1.381)	RPS4 (1.65)	RPS4 (6.43)	ACTB (0.785)	ACTB (1.202)
**5**	GAPDH (1.431)	GAPDH (1.66)	GAPDH (6.66)	GAPDH (0.85)	GAPDH (1.290)
**6**	B2M (1.52)	B2M (1.70)	B2M (7.43)	B2M (0.851)	B2M (1.367)

Stability values obtained by each method are shown in parenthesis for each candidate reference gene. Both Standard deviation (*SD*) and Coefficient of variation (*CV*) rankings are shown for BestKeeper. The genes are ranked from most stable (1) to least stable (6).

**Table 4:** **Intra-group and inter-group variation estimates by NormFinder**

Variation	***ACTB***	***B2M***	***GAPDH***	***RPL17***	***RPS4***	***UBQ***
**Intra-group**	0.491	0.983	0.476	0.631	0.466	0.362
**Inter-group**	0.553	0.635	0.624	0.485	0.392	0.504

Average intra-group and inter-group Cq variation estimates obtained by NormFinder. Groups were constituted by fish of the same age and rearing temperature.

In our analysis*, UBQ* appears ranked first by BestKeeper, GeNorm and comparative delta-Ct method, and ranked second by NormFinder. *B2M*, a frequently used reference gene, is consistently ranked the last by all methods and does not show a strong correlation with any of the other genes (highest correlation 0.619 with *GAPDH*) (Table 3, Figure 2). This gene Cq distribution also deviated from normality. The inconsistency of *B2M* as reference gene has also been previously reported in human tissues [6, 28]. The ranking between these two extremes varies depending on the method.

BestKeeper ranked *UBQ* as the most stable gene with 1.12, a value above the recommended cutoff of 1 [7]. However, considering that our dataset includes samples coming from different tissues (ovary and testes), experimental conditions and development stages, a low standard deviation was not expected. *UBQ* also shows the lowest CV and a high correlation with *RPS4* (r = 0.831). *ACTB* is ranked as the second best reference gene by BestKeeper (SD = 1.21), however, it does not show a high correlation with any of the other genes and it is ranked 4^th^ by other methods (Table 3, Figure 2). A possible explanation for this discrepancy might be the high expression shown by *ACTB* (mean Cq 15.87, more than two cycles higher than any other gene) (Table 1). This renders a lower error when measuring the fluorescence values and a lower copy number difference between samples, which does not imply higher stability between the different experimental conditions. This should be taken into account when choosing a reference gene since they are usually highly expressed genes. The most important characteristic of a reference gene is that its sample-to-sample variation must be representative of the technical error produced by the sampling, extraction and retrotranscription steps in order to reduce target gene Cq values error. Bestkeeper ranks *RPL17* third (SD = 1.46), *RPS4* fourth (SD = 1.65) and *GAPDH* fifth (1.66) (Table 3). An interesting fact is that only *RPS4* shows high correlations with other genes (*UBQ*: r = 0.831, *GAPDH*: r = 0.741 and *RPL17*: r = 0.677) (Figure 2). Like *ACTB* but at the other extreme, *RPS4* shows the lowest amplification cycle (Table 1), which can result in higher technical error. So, according to BestKeeper, *UBQ* is the most stable gene, followed by *ACTB* and *RPL17*. As mentioned, probably *ACTB* is not a good reference gene and it seems that *RPS4* might deserve a better ranking given its correlation with other genes.

NormFinder, which assesses inter-group variation (systematic differences due to age/temperature in our case) in order to discard regulated genes, ranked *RPS4* as the most stable gene (0.613), *UBQ* appears second (0.713) and *RPL17* third (0.721) (Table 3). *RPS4* showed the lowest inter-group variation (0.392), which explains its ranking (Table 4). *GAPDH* is clearly pointed as less stable than *RPS4* and *RPL17* by NormFinder, with an overall score of 0.835 and an inter-group variation of 0.624. Surprisingly, NormFinder suggests *ACTB* and *RPS4* as the most stable two gene combination. *ACTB* is ranked 4^th^ by NormFinder (0.785) (Table 3, Figure 2). The information provided by BestKeeper and NormFinder should be enough to decide among the most stable genes, to say, those with an acceptable low level of overall variation, low inter-group variation and high correlation between them.

The last two methods, comparative delta-Ct method and GeNorm, both follow pairwise approaches but with different procedures and outcomes; the first one ranks the genes following an average pairwise SD while the second follows a progressive exclusion of the least stable gene after pairwise comparison. Both methods agree with the results obtained by NormFinder. GeNorm recommends the couple of reference genes *UBQ*/*RPS4* with a value of 0.952 (the generally accepted cutoff value is 1.50), and points that adding another reference gene (*RPL17*) would not improve normalization (1.154 stability value for *UBQ/RPS4/RPL17*). Pairwise comparison methods tend to select those genes with the highest degree of similarity across the sample set, implying that the candidates with minimal expression variation do not necessarily become top ranked [8]. While both approaches are based in pairwise comparisons, the progressive exclusion of genes by GeNorm increases the tendency to select the most correlated genes.

Since we have obtained inter-group variation estimates by NormFinder (Table 4) which points toward *RPS4, UBQ, RPL17* and possibly *ACTB* not being differentially expressed between groups, and also due to the unexpected presence of *ACTB* in the best normalization indexes calculated by NormFinder, we checked how the use of different normalization factors, geometric mean of different reference genes, affected six sex-differentiation related genes (*CYP19a*, *AMH*, *SOX19*, *SOX9*, *VASA*, *SOX17*) (Table 5). We normalized the samples by [*UBQ]*, [*RPS4*], [*UBQ + RPS4*], [*RPS4 + ACTB*], [*UBQ + RPS4 + RPL17*], *[UBQ + RPS4 + RPL17]* and [*UBQ + RPL17 + RPS4 + ACTB*]. We checked the intragroup and intergroup standard deviation for each of the six target genes and, since the samples were genetically sexed using the SmaUSC-E30 marker according to [22], we also checked the standard deviation of male and female groups (Table 5). This way, we can have an independent measure out of the fish age/rearing temperature groups we have used to check the stability of the reference genes. Interestingly, *UBQ* and *RPS4* seem to behave differently. While *RPS4* renders lower SD values for fish age/rearing temperature groups, *UBQ* normalizes male and female groups better. However, the lowest SD estimates were obtained when both [*UBQ + RPS4*] were used for normalization, except for average intergroup SD where *RPS4* alone performed better. The addition of *RPL17* or *ACTB* in the index for normalization did not yield lower SD estimates. The use of just [*UBQ + RPS4*] for normalization is in agreement with the results of GeNorm and also with the rankings produced by NormFinder and comparative delta-Ct method. The use of [*RPS4 + ACTB*] as suggested by NormFinder does not perform better.

**Table 5:** **Standard deviation for target genes when normalized by different gene combinations**

	Average intragroup SD	Average intergroup SD	Average male group SD	Average female group SD
*UBQ*	1.53	2.83	2.48	2.57
*RPS4*	1.49	2.69	2.50	2.63
*UBQ + RSP4*	1.47	2.71	2.47	2.57
*ACTB + RPS4*	1.53	2.79	2.63	2.8
*UBQ + RPS4 + RPL17*	1.50	2.73	2.53	2.66
*UBQ + RPS4 + ACTB*	1.48	2.78	2.54	2.68
*UBQ + RPS4 + RPL17 + ACTB*	1.50	2.74	2.61	2.78

Intragroup and intergroup normalized Cq standard deviations (SD) averaging the results for the six target genes are shown for Fish age + Rearing temperature groups when normalized by different candidate reference gene combinations. Standard deviations (SD) for males and females when normalized by the same combinations are also shown.

To assess the robustness of each method, we repeated the stability calculations in fifty subsets of the samples (Table 6), 25 subsets include 3 samples of each experimental group (fish age/rearing temperature) and another 25 include 2 samples of each experimental group (a total of 72 and 48 samples per subset respectively). We evaluated the 50 subsets together since the results show similar trends both with three and two samples per group. Since in many studies three genes are used for normalization, we compared not only the whole ranking but also the top3 genes. The most robust method is clearly BestKeeper SD, which renders an identical ranking as that obtained with the whole data set for a 44% of the subsets and, in 40% of the remaining subsets, it ranks the top three genes correctly (a total of 88%). NormFinder selected the same top3 genes also in 88% of the subsets, however the rank order was altered most of the times. On the contrary, the pairwise approaches showed a higher degree of variation, the top3 genes were different from those in the full dataset in 60% of the subsets for comparative delta Ct method and in 66% for GeNorm.

**Table 6:** **Robustness of the gene stability determination method**

	Full dataset ranking comparison	Comparative delta-Ct	Bestkeeper (SD)	NormFinder	GeNorm
3 samples per experimental group subsets	Identical ranking	4	11	1	8
	Top 3 genes in different order	13	11	21	1
	Different ranking	8	3	2	16
2 samples per experimental group subsets	identical ranking	2	11	4	4
	Top 3 genes in different order	11	9	18	4
	Different ranking	12	5	3	17
Total	Identical ranking	12%	44%	10%	24%
	Top 3 genes in different order	48%	40%	78%	10%
	Different ranking	60%	16%	12%	66%

Similarity of 50 subsets stability rankings by each method and the ranking obtained with the whole dataset. 25 subsets are formed by 3 samples per group (age/temperature) and another 25 subsets have 2 samples per group.

### Efficiency determination analysis

We obtained mean gene efficiencies by LingRegPCR, LREanalyzer, Dart and PCR-Miner for each primer pair (Table 7) and correlations between mean efficiencies by each method for each gene (Table 8). There is around a 10% difference between the efficiencies calculated by linear fit methods (LinRegPCR, DART) and non linear fit models (LREanalyzer, PCR-Miner), meaning that exponential methods might be underestimating efficiency or non linear methods overestimating it (or both). Two LREanalyzer efficiency estimates are over 100% (*RPS4* and *UBQ*), which is theoretically impossible for a PCR reaction, so LREanalyzer is likely overestimating qPCR efficiency. However, despite this 10% efficiency difference, mean efficiencies calculated by the four methods are correlated for each gene, indicating that although they are using different algorithms they are rendering similar relative results. Best correlation coefficient and p value are observed between LinRegPCR and PCR-Miner, which might be highlighting the importance of baseline correction since both methods use iterative approaches instead of relying on a fluorescence correction based on the average fluorescence of the first qPCR cycles. Also, LinRegPCR and PCR-Miner include several functions to remove outliers, so filtering the reactions before efficiency calculation might also be important to obtain more precise efficiency estimations.

**Table 7:** **Efficiency values for each gene with each efficiency determination method**

	ACTB	B2M	GAPDH	RPL17	RPS4	UBQ
LREanalyzer	97.82%	98.00%	99.32%	94.46%	100.45%	101.78%
LinRegPCR	87.12%	90.27%	89.24	82.82%	88.61%	89.63%
DART	88.72%	92.62%	89.09%	86.04%	89.39%	90.84%
PCR-Miner	94.42%	99.72%	99.68%	92.23%	98.78%	99.69%

Mean efficiency values for each reference gene with LingRegPCR, LREanalyzer, DART and PCR-Miner.

**Table 8:** **Correlation between efficiency determination methods**

	LREanalyzer	LinRegPCR	DART
LinRegPCR	0.81 (0.052)		
DART	0.6 (0.205)	0.91 (0.013)	
PCR-Miner	0.82 (0.047)	0.94 (0.005)	0.82 (0.047)

Pearson correlation coefficients and p values (in parenthesis) for mean gene efficiencies with each of the four efficiency determination methods are shown.

### Normalization and efficiency correction on target genes

Six target genes (*CYP19a*, *AMH*, *SOX19*, *SOX9*, *VASA*, *SOX17*) involved in gonad differentiation were efficiency corrected and normalized by four different combinations of efficiency determination methods and reference gene combinations (LinRegPCR-*UBQ + RPS4*, LinRegPCR-*B2M*, PCR-Miner-*UBQ + RPS4* and PCR-Miner-*B2M*). For each combination, first, efficiency correction was performed on every Cq value of both reference and target genes. Afterwards, each target efficiency-corrected Cq value was normalized by the reference gene/s efficiency-corrected Cq values, obtaining efficiency-corrected delta Cq values. We computed mean efficiency-corrected delta Cq values and standard deviations for the three temperature groups (high, normal and low temperature) (Table 9) and also for males and females (Table 10). Two different patterns are shown in the tables, one caused by normalization and the other by efficiency correction. A higher standard deviation is obtained in most of the *B2M* normalized dataset compared to the *UBQ + RPS4* normalized ones, which is expected when a gene is not stable. However, this is not true for the *AMH* normal temperature group neither for the gene *VASA,* suggesting some type of co-regulation. The other trend is observed when comparing the LinRegPCR efficiency corrected datasets with the PCR-Miner corrected ones. PCR-Miner produces higher mean Cqs (absolute value) increasing the difference between groups.

**Table 9:** **Efficiency-corrected delta Cqs by temperature group with each efficiency + reference gene combination**

	High T Cq Mean	High T Cq SD	Normal T Cq Mean	Normal T Cq SD	Low T Cq Mean	Low T Cq SD
*CYP19a UBQ + RPS4* LinRegPCR	1.11	4.33	-0.35	3.54	-0.74	5.41
*CYP19a UBQ + RPS4* PCR-Miner	1.19	4.65	-0.38	3.81	-0.8	5.81
*CYP19a B2M* LinRegPCR	0.78	5.26	-0.89	4	0.24	5.8
*CYP19a B2M* PCR-Miner	0.83	5.66	-0.96	4.3	0.26	6.24
*AMH UBQ + RPS4* LinRegPCR	0.07	3.39	1.02	4.47	-1.27	1.48
*AMH UBQ + RPS4* PCR-Miner	0.07	3.65	1.09	4.8	-1.36	1.57
*AMH B2M* LinRegPCR	-0.26	3.67	0.5	4.23	-0.32	2.16
*AMH B2M* PCR-Miner	-0.28	3.94	0.54	4.54	-0.34	2.32
*SOX19 UBQ + RPS4* LinRegPCR	-0.24	3.99	-0.92	3.83	1.33	2.39
*SOX19 UBQ + RPS4* PCR-Miner	-0.26	4.28	-0.98	4.11	1.42	2.56
*SOX19 B2M* LinRegPCR	-0.57	5.19	-1.46	3.97	2.31	3.08
*SOX19 B2M* PCR-Miner	-0.62	5.57	-1.56	4.26	2.48	3.31
*SOX9 UBQ + RPS4* LinRegPCR	0.38	1.82	0.17	1.44	-0.59	1.13
*SOX9 UBQ + RPS4* PCR-Miner	0.4	1.96	0.18	1.55	-0.64	1.21
*SOX9 B2M* LinRegPCR	0.04	2	-0.37	1.75	0.39	2.05
*SOX9 B2M* PCR-Miner	0.05	2.15	-0.4	1.88	0.42	2.21
*SOX17 UBQ + RPS4* LinRegPCR	0.58	2.12	-0.6	1.54	0.09	1.59
*SOX17 UBQ + RPS4* PCR-Miner	0.62	2.26	-0.63	1.65	0.09	1.7
*SOX17 B2M* LinRegPCR	0.25	3.47	-1.13	2.06	1.07	2.39
*SOX17 B2M* PCR-Miner	0.26	3.72	-1.21	2.21	1.15	2.56
*VASA UBQ + RPS4* LinRegPCR	1.26	1.46	-0.96	4.04	-0.19	2.6
*VASA UBQ + RPS4* PCR-Miner	1.35	1.57	-1.03	4.34	-0.21	2.79
*VASA B2M* LinRegPCR	0.93	1.91	-1.5	3.8	0.79	2.46
*VASA B2M* PCR-Miner	0.99	2.05	-1.61	4.08	0.85	2.64

Mean efficiency-corrected delta Cqs and SD values for the three rearing temperatures (T): high, normal and low; in the four datasets produced after efficiency correction with LinRegPCR or PCR-Miner and later normalization with *UBQ* + *RPS4* or *B2M*.

**Table 10:** **Efficiency-corrected delta Cqs by sex group with each efficiency + reference gene combination**

	Female Cq Mean	Female Cq SD	Male Cq Mean	Male Cq SD
*CYP19a UBQ + RPS4* LinRegPCR	-2.82	2.39	4.37	3.17
*CYP19a UBQ + RPS4* PCR-Miner	-3.03	2.57	4.7	3.41
*CYP19a B2M* LinRegPCR	-3.05	2.67	4.74	3.99
*CYP19a B2M* PCR-Miner	-3.28	2.87	5.1	4.29
*AMH UBQ + RPS4* LinRegPCR	0.85	3.24	-1.32	3.54
*AMH UBQ + RPS4* PCR-Miner	0.92	3.49	-1.42	3.8
*AMH B2M* LinRegPCR	0.59	3.26	-0.91	3.69
*AMH B2M* PCR-Miner	0.63	3.51	-0.97	3.95
*SOX19 UBQ + RPS4* LinRegPCR	-2.31	2.54	3.58	1.25
*SOX19 UBQ + RPS4* PCR-Miner	-2.47	2.71	3.84	1.34
*SOX19 B2M* LinRegPCR	-2.54	3.73	3.95	1.59
*SOX19 B2M* PCR-Miner	-2.73	4	4.23	1.72
*SOX9 UBQ + RPS4* LinRegPCR	0.67	1.46	-1.04	0.95
*SOX9 UBQ + RPS4* PCR-Miner	0.72	1.57	-1.12	1.01
*SOX9 B2M* LinRegPCR	0.44	1.72	-0.68	2.07
*SOX9 B2M* PCR-Miner	0.47	1.85	-0.73	2.23
*SOX17 UBQ + RPS4* LinRegPCR	-0.98	1.1	1.53	1.62
*SOX17 UBQ + RPS4* PCR-Miner	-1.05	1.17	1.63	1.73
*SOX17 B2M* LinRegPCR	-1.22	2.35	1.89	2.39
*SOX17 B2M* PCR-Miner	-1.31	2.52	2.03	2.57
*VASA UBQ + RPS4* LinRegPCR	-0.17	3.2	0.27	2.87
*VASA UBQ + RPS4* PCR-Miner	-0.19	3.44	0.29	3.08
*VASA B2M* LinRegPCR	-0.41	3.09	0.63	2.98
*VASA B2M* PCR-Miner	-0.44	3.32	0.68	3.2

Mean efficiency-corrected delta Cqs and standard deviation (SD) values for males and females in the four datasets produced after efficiency correction with LinRegPCR or PCR-Miner and later normalization with *UBQ* + *RPS4* or *B2M*.

Furthermore, the use of a gene which presents systematic differences between groups for normalization can lead to changes in the mean Cq values of some genes. For example, *AMH* gene expression in each temperature group is severely affected by normalization with *B2M*, varying from 0.07 to -0.26 at high temperature (when compared to normalization by *UBQ* + *RPS4*), from 1.02 to 0.5 at normal temperature and from -1.27 to -0.32 at low temperature (LinRegPCR values).

## Discussion

### Reference gene analysis

The four methods commonly used to check the stability of reference genes, comparative delta-Ct method, NormFinder, BestKeeper and GeNorm, represent viable strategies, although none of them is currently considered the best one and some problems can arise in certain experimental scenarios. The BestKeeper method is apparently the “common sense” solution to measure stability since standard deviation is a direct measure of variation. However, a gene might show a low standard deviation but still not be a good reference gene if its variation does not reflect the errors produced by sampling, RNA extraction and retrotranscription steps. This problem could be circumvented by analyzing the correlations between genes, assuming that the reference genes are not co-regulated. This means that sampling point differences (time and temperature in our experiment) affecting one of the genes should not affect the others, and so the correlations between them would reflect the inter-sample variation produced by the sample processing steps and not by co-regulation due to the experimental conditions. Nevertheless, it is risky to assume that genes are not co-regulated because this cannot be easily demonstrated. The GeNorm and the comparative delta-Ct method approaches present the same problem but in addition these methods rank genes mainly by their correlations, to say, GeNorm establishes the most stable genes by assuming “that the control reference genes are not co-regulated” [3], and the same happens to the comparative delta-Ct method which follows a very similar approach. As a consequence, two co-regulated genes could fully spoil the analysis leading to wrong reference genes. Finally, NormFinder is not affected by the co-regulated gene drawback since it takes into account intergroup variation (finding genes which do not vary depending on time or temperature in our case), which should be as lower as possible for a good reference gene; however, similarly to BestKeeper, a low overall intergroup and intragroup variation does not necessarily mean that it is a good reference gene. The advantages and disadvantages of each strategy should be taken into account when analyzing putative reference genes according to the experimental scenario.

NormFinder and GeNorm are the most extended methodologies to find the optimum reference genes. In many cases, NormFinder and GeNorm algorithms render very similar results, however, discrepancies between the output of NormFinder and GeNorm have been previously described [9, 29, 30]. In these works, the CV has been used to decide which genes should be used for normalization, confirming NormFinder results in every case. While NormFinder results are non-biased, GeNorm stepwise exclusion can lead to awkward results by selecting reference genes which in fact are not the most stable. NormFinder, BestKeeper and comparative delta-Ct stability method results have also been reported to be more consistent among them than with those of GeNorm [30], although in other study BestKeeper was reported as the least consistent method [31]. Our results with the whole dataset support the high consistency between NormFinder and comparative delta-Ct method, while BestKeeper results seem to be the least consistent and only correlation values between reference genes seem to suggest a similar ranking.

However, when working with different subsets which include a lower number of samples, the pairwise approaches results vary significantly between subsets. This lack of robustness has been described previously: it was shown that the exclusion of a single sample could change the status of one gene from unstable to 2^nd^ most stable gene by GeNorm [29]. GeNorm lack of robustness can most likely be explained by the removal of the least correlated gene by pairwise comparison with all the others until only two genes are left, which can lead to stable genes being removed of the analysis early on. Robustness is a critical parameter. Since experiments are budget limited, it is important to be able to determine correct reference genes with a low number of qPCR reactions. BestKeeper and NormFinder appear to be more robust than comparative delta-Ct method and GeNorm in our study.

There is not a method to check how much normalization has improved our gene expression data. In principle, a reduction in the Cq variability of the gene of interest should be expected, however the highest reduction of this variability would also occur if the gene of interest and the reference gene(s) are co-regulated, so this is a risky strategy. An example of this is observed in *VASA* (and one *AMH* group) standard deviation after normalization, obtaining a lower SD when normalized by a clearly not stable gene (*B2M*) than when normalized by *UBQ* + *RPS4*, suggesting co-regulation between *VASA* and *B2M*. The same applies to detecting significant/non-significant results depending on the reference(s) gene(s) used for normalization. This is only useful to stress the importance of choosing a good reference gene, not to choose between one or another since a co-regulated reference gene would lead to non-significant results even if there are expression differences between groups.

The fact that there is not a post-control which enables us to check if we have chosen the correct reference gene makes the choice even more critical. Every experiment and dataset is different, so the analysis has to be done carefully. Given the huge importance of normalization and its great impact on the conclusions, it would be recommended to analyze each case separately, paying attention to details; using any method as a black box can lead up to low confident results. Several studies have solved the disagreement between the four methods by ranking them according to the geometric mean of the four ranking numbers for each gene, the lower the mean a gene gets the most stable it is [30]. However, attributing the same weight to every method is arguable, especially because some of these methods include redundant information. This is a practical option without any biological meaning. If the four methods disagree, we recommend instead relying on the ranking provided by NormFinder, while ignoring its suggested combination, supported by descriptive statistics like mean, standard deviation and correlations, information offered by BestKeeper or any common statistical package. This approach would enable to assess the two most important and complementary issues: absence of inter-group variation and correlation between reference genes. This approach does not make any previous assumption and has proven to be robust when only a few samples are assayed.

To our knowledge this is the first experiment to analyze the stability of reference genes during the gonad development in fish. Even in mature organs, there is only one study carried out in zebrafish were testis and ovaries were analyzed separately [32]. However, studies have been carried out in other organs. The stability of several genes was studied in the liver, spleen, kidney, heart, brain, gill and muscle of turbot subjected to *Edwardsiella tarda* infection [5]. Gene stability was checked before infection in all the organs together by NormFinder and GeNorm. In that study, out of eight genes, NormFinder ranked *RPSD* as the most stable one, followed by *ACTB*, *RPL17*, *B2M* and *GAPDH* among those genes shared with our study, although primer pairs for GAPDH were different. We tried to develop primers for *RPSD* but they were discarded due to late amplification cycle in gonad (>25). *UBQ* and *RPS4*, were not assayed in that work. *GAPDH*, which has been classically used as a reference gene but recently classified as unstable in several studies [3, 4, 9, 28], performed badly in both our study and [5]. However, *GAPDH* is ranked as the most stable gene in heart and liver in [5], which emphasizes the importance of checking reference gene stability in each study separately, reference genes cannot be “exported”. There are two *GAPDH* isoforms in diploid teleost fish as a result of the fish-specific genome duplication event, however the same variant has been analyzed in both studies (*GAPDH-2*).

There are two similar qPCR studies carried out in flatfish. The first one studied six reference genes during *Hippoglossus hippoglossus* development in sixteen different tissues using BestKeeper, NormFinder and GeNorm [26]. Gonads were not included. They assayed *ACTB* which was found as one of the least stable genes. The most stable genes found were *EF1a1* and *RPL7*. The second study was carried out during larval development in *Solea senegalensis* and *Hippoglossus hippoglossus*. The stability of twelve genes, including *UBQ*, *RPS4*, *ACTB* and *GAPDH-2*, was checked by GeNorm and NormFinder [27]. The combined stability index of the two species ranked *UBQ*, *RPS4* and *eEF1a1* as the three best normalization genes by both methods, while *ACTB* appears in 5^th^ and 6^th^ place, respectively. Interestingly, in *Solea senegalensis, GAPDH-2* appears ranked 3^rd^ and 1^st^ by GeNorm and NormFinder, respectively.

### Efficiency determination analysis

Efficiency determination is an essential step in qPCR. Constant amplification efficiency in all compared samples is a very important criterion for reliable comparison between samples. It is also crucial for an accurate quantification of gene expression. Ideally, the efficiency of an assay should be 100%, which means that during the logarithmic phase of the reaction the PCR product is doubling each cycle.

Each of the four tested efficiency determination methods differ in their baseline fluorescence determination, type of fit to the log-linear phase of the qPCR reaction, and preprocessing steps to remove outliers. DART [10] is based on a linear regression of the exponential phase of the qPCR reaction. Baseline subtraction is determined by fitting a saturation function to the first 2-10 cycles of the qPCR reaction. Then, a linear regression is performed in a 10-fold range around the middle point of the exponential phase, which is calculated using the maximum fluorescence and standard deviation of the fluorescence in the first 10 cycles. LinRegPCR [14] is also based on a linear regression fit to the log-linear phase of the amplification curve. LinRegPCR determines baseline fluorescence through an iterative algorithm to get the best fit of the linear regression to 4-6 points in the log-linear phase of the reaction. Then, after baseline subtraction, these points are used for efficiency determination. PCR-Miner [16] uses a non-linear regression fit. As LinRegPCR, baseline fluorescence is determined by an iterative fit to a four-parameter logistic model which also determines the exponential phase of the reaction. Then, a three-parameter exponential model is fitted to the exponential phase to determine efficiency. Finally, LREanalyzer (linear regression of efficiency) [15] uses a sigmoidal fit approach. Baseline subtraction is determined by averaging 6-12 cycles fluorescence values. Then, efficiency estimates are calculated for each cycle of the qPCR reaction. An LRE window is selected with those cycle efficiencies which fit to a linear regression. Finally, a derivative of the Boltzmann sigmoidal function is used for the calculation of the maximum efficiency of the reaction.

Logistic models are pure empirical models not designed to be kinetically realistic [33] and rely purely in their good fit to the real-time PCR curve. As previously reported by other study, qPCR curves are not symmetric since they do not have the same curvature at both sides of the inflection point, implying the existence of two or more different mechanisms affecting the efficiency of the reaction [1] and so, making this good-fit models hardly reliable. Furthermore, in some reactions SYBR green depletion might be the main mechanism leading to the plateau phase of the curve [34]. While SYBR green has an impact in the visualization of the real-time reaction, it does not have a connection with the kinetics of the PCR reaction.

A recent qPCR study has tried a new approach to assess qPCR efficiency, defining the global efficiency as the sum of denaturing efficiency, annealing efficiency, polymerase binding efficiency and elongation efficiency [13]. The polymerase binding efficiency and the elongation efficiency can be constant provided that there is an excess of polymerase and a long elongation time. However, the denaturing efficiency is constantly decreasing each cycle at the same rate due to thermal damage in both the DNA and the polymerase. The annealing efficiency is also decreasing and depends on the proportion of ssDNA bound to the primers during this step and total ssDNA present. Some ssDNA chains might bind to its complementary strand instead of to the primers [13]. This efficiency varies from cycle to cycle. This theoretical study was validated in [35]. So, at first, a constant efficiency should not be assumed. Still, the qPCR curve shows a large exponential component, since, as confirmed by a previous study, in most cases the best fit to the log-linear region of the qPCR reaction is exponential [1], suggesting that before and at the log-linear region the qPCR efficiency reduction is low.

A recent study analyzed all publicly available efficiency determination methods [36] in a large dataset, included four-point 10-fold dilution series, which allows calculation of the bias of each method. Similarly to our results, they report LinRegPCR and DART to produce an underestimation of efficiency and PCR-Miner and LRE analyzer an overestimation. They also analyze different parameters and find LinRegPCR and PCR-Miner amongst the best methods for most of the evaluated characteristics, for example precision and resolution, performing better than LREanalyzer and DART. The reader is encouraged to consult [36] to learn more about the different efficiency determination methods, their characteristics and performance differences. This study [36] is the most complete on qPCR efficiency determination methods done so far.

Both LinRegPCR and PCR-Miner performed similarly and produced highly correlated efficiency estimates in our study. The main difference was that while LinRegPCR underestimates efficiency, PCR-Miner overestimated it. Knowing this, LinRegPCR is probably the best choice for the average qPCR researcher since it will not produce erroneous significant differences between groups (false positives) or an overestimation of the fold change. However, LinRegPCR might not be the best option for clinical purposes, where the method of choice should be considered depending on the consequences of a false positive/overestimation or a false negative/underestimation. There are a good number of alternative efficiency estimation algorithms, however they are implemented as extensions to the open source statistical programming environment R [37] and probably not available for most researchers, so they have not been analyzed here. Still, LinRegPCR and PCR-Miner perform as well or better than all other methods, as shown in [36].

As a final remark, although [36] clearly improved our understanding of the different efficiency determination methods available, currently there is not a clear best method for estimating qPCR efficiencies. However, the publication of a theoretical study based on the PCR kinetics which defines the overall PCR efficiency as the product of the efficiency of each of the separate steps [13] and its experimental validation [35] is a good step towards finding a biologically meaningful solution. Similar approaches will be likely applied in more studies in the near future provided they are implemented in appropriate user friendly softwares for the whole research community.

### Normalization and efficiency correction on target genes

The effects of normalization with wrong genes are important; a high standard deviation will produce higher p values and, so, possibly lead to missing biologically relevant differences. Even worse, the use of a regulated gene (which shows systematical differences between experimental groups) for normalization, will lead to changes in gene values which can end in misguided results. The effect of efficiency correction, though not so dramatic, is also important since it can lead to overestimation (or underestimation) of differences between groups.

## Conclusions

We found the ranking produced by NormFinder method as the most reliable one to choose reference genes for qPCR analysis when results differ between gene stability determination methods. NormFinder information should be complemented by the descriptive statistics offered by BestKeeper, especially the correlation coefficient. Accordingly, we found that *UBQ* and *RPS4* should be used as reference genes to study turbot gonad development from 30 up to 135 days post fertilization. We found pair-wise methods to be less robust than NormFinder and BestKeeper and also the suggested NormFinder two genes combination not reliable. We also recommend the use of LinRegPCR for efficiency determination for research purposes, however, efficiency determination is still a matter of discussion and probably new improved models will be published in the upcoming years.

## Methods

### Rearing conditions and sampling

Turbot fertilized eggs were obtained by crossing one female with two males and reared in tanks at the Instituto Oceanográfico de Vigo at three different temperatures (15°C, 18°C and 23°C). The samples were taken at the following stages: 30, 45, 60, 75, 90, 105, 120 and 135 days post fertilization (dpf). At each sampling point 10 individuals were taken per temperature (3×10) and their gonads excised as accurately as possible. The final number of samples tested was 240: eight different developmental stages, thirty gonad samples per stage (ten per each temperature). Samples were immediately embedded in RNAlater for preservation (Qiagen, Valencia, CA). Male and female gonads can be differentiated at 90dpf by histology (Cal R, Lluch N, Martínez P. Gonadal sex differentiation in turbot (*Scophthalmus maximus*). In preparation) and microarray (Ribas L, Robledo D, Viñas A, Martínez P, Piferrer F. Transcriptomic study of the sex differentiation process in turbot. In preparation). Also, *cyp19a* raw expression values by qPCR can perfectly distinguish females from males starting at 105 dpf (Additional file 2: Figure S1).

Animals were treated according to the Directive 2010/63/UE of the European Parliament and of the Council of 22 September 2010 on the protection of animals used for experimentation and other scientific purposes. All experimental protocols were approved by the Institutional Animal Care and Use Committee of the University of Santiago de Compostela (Spain).

### RNA isolation and cDNA synthesis

Total RNA was extracted by homogenization in TRIZOL (Invitrogen, Paisley, UK) following the manufacturer’s protocol. Total RNA was treated with RNase-free Recombinant DNase I(Roche Diagnostics, Mannheim, DE) and RNA concentration was assessed by spectrophotometry and its quality checked using an Agilent 2100 Bioanalyzer (Agilent Technologies, Santa Clara, US). Total RNA (1.2 μg) was reverse transcribed by random primers using AffinityScript Multiple Temperature cDNA Synthesis Kit (Agilent Technologies) following the manufacturer’s protocol and then diluted 1:2 with nuclease-free water.

### Real-time PCR

Real-time PCR was performed on a Stratagene Mx3005P (Agilent Technologies) thermocycler using Brilliant III Ultra-Fast SYBR Green qPCR Master Mix in a final volume of 12.5 μL following the manufacturer’s protocol with 1 μL of cDNA per reaction. Gene-specific primers for the reference genes *RPL17* (Ribosomal Protein L17), *B2M* (Beta-2-microglobulin) and *ACTB* (beta-actin) were obtained from [5] and primers for *UBQ* (Ubiquitin), *RPS4* (Ribosomal Protein S4) and *GAPDH* (glyceraldehyde-3-phosphate dehydrogenase) were designed in our laboratory (Table 11). Specificity for each primer pair was first checked by melting curve profile and then confirmed by PCR product sequencing. *RPL17*, *B2M* and *ACTB* were chosen as putative reference genes because they were among the most stable genes in a previous study using different tissues in turbot [5], while *UBQ*, *RPS4* and GAPDH were chosen because of their general use in many studies in other species and proved to be stably expressed in a microarray study carried out in our laboratory (unpublished data). Gene specific primers were also designed in our laboratory for six target genes involved in sex differentiation (*CYP19a, AMH, SOX9, SOX19, SOX17 and VASA*) (Table 11), and amplification was performed following the same procedure. Primer concentration was 300 nM and each sample was run in duplicate. The cycling parameters were: 50°C for 2 min, 95°C for 10 min, followed by 40 cycles of amplification at 95°C for 15 sec and 60°C for 1 min. Finally, a dissociation step was performed after amplification to ensure the presence of a single amplification product. All the samples (240) were assayed for each gene. A sample maximization strategy was carried out, meaning that as many samples as possible were run in a single plate, and so, each gene was tested in the minimum amount of plates as possible. In every PCR plate, non-template controls were included to confirm the absence of contamination. In addition, three samples (interplate calibrators) were run in triplicate in every plate in order to correct inter-assay variation, each Cq value in a plate was corrected by adding or subtracting the difference between interplate calibrators mean value in the plate and their overall mean value for all the plates [38]. Real-time PCR data were obtained by the MxPro software (Agilent Technologies) and quantification cycle values (Cq) calculated for each replicate and then averaged to obtain the final Cq value. Cq determination fluorescence threshold was the same for the six genes, a background-based threshold was determined for the six genes separately and the highest one applied for the six genes.

**Table 11:** **Primer table**

Gene name	Accession ID	Primer F (5’ - > 3’)	Primer R (5’ - > 3’)	Product Length (bp)
*RPS4*	FE943956	CAACATCTTCGTCATCGGCAAGG	ATTGAACCAGCCTCAGTGTTTAGC	143
*RPL17*	DQ848879	ACCAGTGCGTCCCCTTCA	CTCATCTTCGGAGCCTTGTTC	214
*GAPDH*	FE950888	CGCCCATAGCCCAGTCATAGC	TGGCAGAGGGAGGTGGAGAG	167
*ACTB*	EU686692	GTAGGTGATGAAGCCCAGAGCA	CTGGGTCATCTTCTCCCTGT	204
*UBQ*	FE946708	GCGTGGTGGCATCATTGAGC	CTTCTTCTTGCGGCAGTTGACAG	124
*B2M*	DQ848854	CTCTGGCTGTTTTCGTCTGCT	TCCTTTCCGTTCTCTCCCG	86
*CYP19a*	JQ403643	CAGCGAGGAAGCTGGCAAACA	ACACGCAGACTCGGCTTTTTACATC	148
*AMH*	JQ403642	CCAGGGCGGACCCCGATAAC	TGGCTGTGTTTGGACCCACGAG	99
*SOX9*	JQ300535	ATCAGTACCCACACCTGCATAAC	TCAGCCTCCTCCACGAACG	103
*SOX19*	JQ403639	ACCGAGCGGTTTGTGCCTTG	TCCTCTGGATGCAGTGCTGATTGT	122
*SOX17*	JQ403638	TGTTCGGGAAGCAGGTGAAAGGT	CTTGTTGCCATTTTAGGGGACAGT	92
*VASA*	JX235364	CTTAGCTGTGGGCGTGGTGGG	ACGTTCTCCTGGCACATCAACG	190

Gene name, accession number, primer sequences and amplicon size of the reference genes (*RPS4, RPL17, GAPDH, ACTB, UBQ, B2M*) and the target genes (*CYP19a, AMH, SOX9, SOX19, SOX17, VASA*) are shown.

### Reference gene analysis

A total of six reference genes were selected for gene expression analysis in turbot gonad (Table 1). Their stability was analyzed with the comparative delta-Ct method [6], BestKeeper [7], GeNorm [3] and NormFinder [8], which use different approaches to establish gene stability, but in all of them, the lower the value the more stable the gene is. R program v. 3.0.2 [37] with the packages “psych”, “gclus” and “fBasics” was used for other statistic operations and graphic generation. Comparisons between methods were performed with the whole data set and also with subsets of samples. We compare 25 subsets with 3 samples per experimental group (72 samples in a total of 24 groups) and 25 subsets with 2 samples per experimental group (48 samples in a total of 24 groups) to assess robustness of each method. Furthermore, six target genes involved in sex differentiation were subjected to normalization by different reference gene combinations.

### Efficiency analysis

Efficiency of each primer pair was checked for each reference gene by four different methods: LinRegPCR [14], LREanalyzer [15], DART [10] and PCR-Miner [16]. Each method calculates individual efficiency values for each qPCR reaction and then, these are averaged to obtained mean efficiency values for each gene. Raw fluorescence values (without baseline correction) were used as input for each efficiency determination method.

### Normalization and efficiency correction on target genes

Efficiency corrected Cq values by LinRegPCR and PCR-Miner were obtained for the six target genes, following the formula “efficiency-corrected Cq = Cq * (log(E) / log(2))” [38]. These corrected Cqs were then normalized by *UBQ* + *RPS4* and *B2M* and then mean centered. This produced four datasets. Mean and standard deviation were obtained for temperature (high, normal and low) and sex (male and female) groups for each gene in each dataset.

## Electronic supplementary material

Additional file 1: Tables S1 and S2: Degree-days group equivalences. Description: Table S1, the equivalence between the days post fertilization – temperature groups and degree-days is shown. Table S2, normfinder results for days post fertilization – temperature and degree-days groups are shown. (PDF 437 KB)

Additional file 2: Figure S1: *Cyp19a* expression levels at 105 dpf. Description: *Cyp19a* mean centered Cq values in the gonads of turbot at 105 days post fertilization. High expression is observed in females and low expression in males. (PNG 14 KB)
