# Supplementary material for: Analysis of qPCR reference gene stability determination methods and a practical approach for efficiency calculation on a turbot (Scophthalmus maximus) gonad dataset
Source: BMC Genomics. 2014 Aug 4;15(1):648. doi: 10.1186/1471-2164-15-648 (PMC4133071; doi:10.1186/1471-2164-15-648)
Supplement: Supplementary file 1 — Additional file 1: Tables S1 and S2: Degree-days group equivalences. Description: Table S1, the equivalence between the days post fertilization – temperature groups and degree-days is shown. Table S2, normfinder results for days post fertilization – temperature and degree-days groups are shown. (PDF 437 KB) [file 12864_2013_6341_MOESM1_ESM.pdf]

Table S1: The equivalence between Age+Temperature groups and degree-days is shown. Two groups (75dpf+18ºC and 90dpf+15ºC) would merge if we followed the "degree days" criterion, going from 24 to 23 groups.

		**Temperature**		
		15	18	23
**Days post fertilization**	30	450	540	690
	45	675	810	1035
	60	900	1080	1380
	75	1125	1350	1725
	90	1350	1620	2070
	105	1575	1890	2415
	120	1800	2160	2760
	135	2025	2430	3105

Table S2: Normfinder results for days post fertilization – temperature and degree-days groups are shown. We repeated NormFinder calculations for reference gene stability determination merging the two groups which would be grouped according to degree-days (75dpf+18ºC and 90dpf+15ºC) and compared them to the results shown in the manuscript for gene stability by NormFinder (the other methods for reference gene stability or efficiency determination are independent of grouping). The best suggested combination of two genes was ACTB and RPS4 for both. As can be seen in the table, the ciphers vary but the classification remains the same and so the conclusions of the manuscript would be unaffected if we changed grouping.

**Rank**	**NormFinder**	**NormFindes (Degree days)**
**1**	RPS4 (0.613)	RPS4 (0.619)
**2**	UBQ (0.713)	UBQ (0.698)
**3**	RPL17 (0.721)	RPL17 (0.707)
**4**	ACTB (0.785)	ACTB (0.781)
**5**	GAPDH (0.85)	GAPDH (0.844)
**6**	B2M (0.851)	B2M (0.856)
