# Supplementary figures and images for: Analysis of qPCR reference gene stability determination methods and a practical approach for efficiency calculation on a turbot (Scophthalmus maximus) gonad dataset
Source: BMC Genomics. 2014 Aug 4;15(1):648. doi: 10.1186/1471-2164-15-648 (PMC4133071; doi:10.1186/1471-2164-15-648)

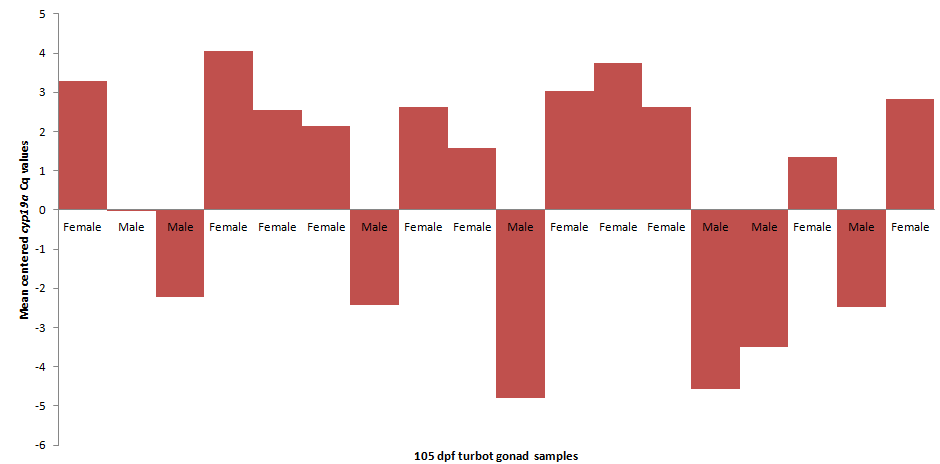

Supplement: Supplementary file 2 — Additional file 2: Figure S1: Cyp19a expression levels at 105 dpf. Description: Cyp19a mean centered Cq values in the gonads of turbot at 105 days post fertilization. High expression is observed in females and low expression in males. (PNG 14 KB) [file 12864_2013_6341_MOESM2_ESM.png]
